# Predictors of the need for airway adjuncts during mask ventilation in Thai adult patients undergoing general anesthesia: A prospective observational study

**DOI:** 10.1371/journal.pone.0359773

**Published:** 2026-10-06

**Authors:** Orarat Karnjanawanichkul, Chanikarn Nuanthong, Ngamjit Pattaravit, Wirat Wasinwong, Sawitree Sricharoenpongchai, Anongluk Samakkee

**Affiliations:** Department of Anesthesiology, Faculty of Medicine, Prince of Songkla University, Songkhla, Thailand; Sapienza University of Rome: Universita degli Studi di Roma La Sapienza, ITALY

## Abstract

**Background:**

Mask ventilation is a cornerstone of airway management during general anesthesia, yet the need for airway adjuncts during mask ventilation remains poorly predicted by current preoperative assessments. We aimed to identify independent clinical predictors of this outcome in Thai adults undergoing elective surgery.

**Methods:**

This single-center prospective observational study included 266 adults undergoing elective general anesthesia at a university hospital in Thailand between September 2018 and October 2019. Preoperative airway assessment included Mallampati classification, dentition status, neck circumference, history of snoring or obstructive sleep apnea (OSA), and other standard variables. The primary outcome was the need for airway adjuncts or optimization during mask ventilation, defined as Han grade ≥2. Han grade ≥2 was selected because only 10 grade-3 events occurred, yielding an events-per-variable ratio of 2.5 under the grade ≥3 definition — insufficient for reliable multivariable regression. Independent predictors were identified using multivariable logistic regression with backward stepwise elimination. Discriminative ability was evaluated using receiver operating characteristic (ROC) analysis and area under the curve (AUC) for both neck circumference alone and the complete four-variable model. Bootstrap internal validation (1,000 resamples) was performed by repeating the model-building procedure, including univariable screening and backward stepwise elimination, within each resample.

**Results:**

The need for airway adjuncts (Han grade ≥2) occurred in 48 patients (18.0%); 38 (14.3%) had Han grade 2 and 10 (3.8%) had Han grade 3. Four variables were independently associated with the need for airway adjuncts in the multivariable logistic regression analysis: snoring or OSA (aOR 5.27, 95% CI 1.94–14.35), absence of ≥2 upper incisors (aOR 2.30, 95% CI 1.08–4.89), neck circumference ≥35 cm (aOR 3.02, 95% CI 1.42–6.44), and Mallampati class III–IV (aOR 2.47, 95% CI 1.22–4.97). The final four-variable model had an apparent AUC of 0.804 (95% CI 0.745–0.862). Bootstrap internal validation with 1,000 resamples, repeating the model-building procedure within each resample, yielded a mean optimism of 0.040 and an optimism-corrected AUC of 0.764 (95% bootstrap interval 0.708–0.820). The Hosmer–Lemeshow goodness-of-fit test showed no evidence of poor model fit (P = 0.077). A simple bedside integer risk score (0–5 points) had an AUC of 0.801. At an exploratory cutoff of ≥3, the sensitivity was 0.85, specificity 0.63, PPV 0.34, and NPV 0.95.

**Conclusion:**

Snoring or OSA, neck circumference ≥35 cm, Mallampati class III–IV, and absence of ≥2 upper incisors were independently associated with the need for airway adjuncts during mask ventilation in Thai adult patients. All four predictors can be assessed at the bedside without additional equipment, suggesting their integration into routine preoperative airway evaluation. As a single-center derivation study, these findings are hypothesis-generating and require external validation in independent Southeast Asian cohorts before clinical adoption.

## Introduction

Mask ventilation is a cornerstone of airway management during general anesthesia and often the first line of rescue when tracheal intubation fails [1]. When mask ventilation itself is inadequate, hypoxemia can develop rapidly, and airway-related complications remain among the most serious contributors to anesthetic morbidity and mortality [2,3].

Preoperative airway assessment is a standard part of anesthetic practice, and current guidelines consistently stress the need to anticipate difficulty in both ventilation and intubation [4,5]. Yet most of the published evidence has concentrated on difficult laryngoscopy and intubation; predictors specific to difficult mask ventilation are far less well characterized [6,7].

A number of risk factors have been proposed, including obesity, absence of ≥2 upper incisors, higher Mallampati class, and increased neck circumference [8–10] Results across studies, however, have been inconsistent [7], and meta-analyses confirm that no single bedside test is sufficiently accurate on its own — for example, a large meta-analysis found that the modified Mallampati test had poor-to-moderate discrimination for predicting difficult intubation [11], and a Cochrane systematic review similarly found limited value of bedside airway examination tests [12] — pointing to the value of combining multiple assessments [4]. Anthropometric variables—especially neck circumference and obesity-related changes in upper airway anatomy—have attracted growing attention in recent work [5,8,9].

Despite these advances, nearly all available data come from Western populations. Evidence from Southeast Asia is scarce. Asian populations differ from Western cohorts in several anthropometrically relevant ways: lower average BMI at equivalent levels of upper airway adiposity, differences in craniofacial anatomy, and distinct patterns of fat deposition around the neck and pharynx [13,14]. These differences suggest that predictive thresholds for continuous variables such as neck circumference, derived from predominantly Caucasian cohorts, may not translate directly to Southeast Asian patients.

We therefore set out to identify independent predictors of the need for airway adjuncts during mask ventilation among Thai adults undergoing elective surgery under general anesthesia.

## Methods

### Study design

This prospective observational study was conducted in accordance with the Strengthening the Reporting of Observational Studies in Epidemiology (STROBE) guidelines. Ethical approval was obtained from the Human Research Ethics Committee of the Faculty of Medicine, Prince of Songkla University, Songkhla, Thailand, on June 28, 2018 (approval number: REC 61-109-8-1). Written informed consent was obtained from all participants before enrollment.

### Study setting and participants

The study was carried out at Songklanagarind Hospital, Thailand, between September 2018 and October 2019. Adult patients (≥18 years) scheduled for elective surgery under general anesthesia with planned airway management using either endotracheal intubation or a supraglottic airway device were considered eligible. Patients were excluded if they had known or obvious airway abnormalities (such as facial deformity or neck mass), a documented history of difficult airway, a pre-existing tracheostomy or endotracheal tube, or required rapid sequence induction.

### Preoperative assessment

Preoperative airway evaluation was performed in all patients by a single anesthesiology resident in order to reduce interobserver variability.

The following variables were recorded: age, sex, body mass index (BMI), and ASA physical status. Airway assessment included Mallampati classification, upper lip bite test (ULBT), inter-incisor distance, thyromental distance, sternomental distance, neck circumference, and cervical mobility (flexion and extension). The presence of beard, dentition status (defined as absence of ≥2 upper incisors), and a history of snoring or obstructive sleep apnea (OSA) were also documented. Snoring and OSA were recorded as a single combined dichotomous variable, ascertained by self-report using a single question, without a validated screening instrument or polysomnography confirmation.

### Intraoperative management and outcome assessment

Anesthetic management was conducted according to routine clinical practice. Following induction of anesthesia, mask ventilation was performed by the attending anesthesiologist, resident, or nurse anesthetist, depending on the clinical setting. Han grade was assigned by the primary ventilating provider at the time of mask ventilation (self-grading); no independent observer was used for outcome ascertainment.

The difficulty of mask ventilation was graded using Han classification (Table 1).

**Table 1:** Han grading scale for mask ventilation.

Grade	Description
1	Easy ventilation
2	Ventilation requiring an oral airway or adjunct
3	Difficult ventilation requiring two providers
4	Unable to mask ventilate

The primary outcome was the need for airway adjuncts or additional optimization during mask ventilation, defined as Han grade ≥2. This threshold was used because only 10 patients (3.8%) had grade 3 and none had grade 4, providing insufficient events for reliable multivariable analysis using the conventional Han grade ≥3 definition. Previous studies [7,8] used conceptually similar definitions of difficult mask ventilation that included the need for airway adjuncts or additional maneuvers, although neither study explicitly applied the Han grade ≥2 threshold. The Han grade ≥2 threshold used in the present study captures clinically relevant events requiring an airway adjunct or additional optimization. Nevertheless, this definition is broader than the grade ≥3 threshold commonly used to define difficult mask ventilation, which should be considered when comparing incidence estimates across studies.

Adequate ventilation was defined as the presence of visible chest rise, minimal mask leak, oxygen saturation ≥90%, an acceptable capnography waveform, and airway pressure ≤35 cmH_2_O.

### Sample size calculation

Sample size was initially estimated using the formula for a single proportion. Previous studies reported a wide range in the incidence of difficult mask ventilation (0.7%–15%), which was considered too variable for direct use. Therefore, a pilot dataset was used, yielding an estimated incidence of 11.1%.

Based on this estimate, with a two-sided alpha of 0.05 and a maximum tolerated error of 0.10, the minimum sample size required to estimate incidence was 38 patients. After accounting for potential dropouts and incomplete data, this was increased to 53 patients.

However, as the primary objective was to identify predictors using multivariable logistic regression, the final sample size was determined based on the events-per-variable approach. Assuming at least 10 outcome events per variable and aiming to evaluate up to four key predictors, a total sample size of 295 patients was targeted; 266 patients were ultimately enrolled within the study period. The final enrolled sample yielded 48 outcome events, providing an EPV of 12:1 for the four-predictor final model, which meets the recommended minimum of 10.

### Statistical analysis

Data were analyzed using STATA software (StataCorp, College Station, TX, USA). Normality of continuous variables was assessed using the Shapiro-Wilk test and visual inspection of Q-Q plots. Normally distributed variables are presented as mean ± standard deviation (SD) and compared using Student’s t-test after confirming equality of variances with Levene’s test; non-normally distributed variables are presented as median (interquartile range, IQR) and compared using the Wilcoxon rank-sum test. Categorical variables are expressed as counts and percentages and compared using the chi-square or Fisher’s exact test, as appropriate.

Han grade ≥3 was examined as a sensitivity outcome. Because only 10 patients met this threshold, reliable multivariable analysis was not feasible; therefore, the sensitivity analysis was restricted to descriptive reporting.

For the primary outcome of Han grade ≥2, variables with P < 0.05 in univariable analysis were entered into the initial multivariable logistic regression model. Backward stepwise elimination was then applied, retaining variables with P < 0.05 in the multivariable model. We acknowledge that this approach to variable selection introduces selection bias and may exclude variables with true predictive value that were masked in univariable analysis. Multicollinearity among retained predictors was assessed using the variance inflation factor (VIF). Results are reported as adjusted odds ratios (ORs) with 95% confidence intervals (CIs). Missing data were not imputed. All analyses were performed on complete cases.

The discriminative ability of neck circumference and the complete four-variable model were assessed using receiver operating characteristic (ROC) curve analysis. The area under the curve (AUC) was calculated, and the 95% confidence interval for the AUC was estimated using the DeLong method. A cohort-derived cutoff for neck circumference was selected based on the balance between sensitivity and specificity.

Bootstrap internal validation with 1,000 resamples was performed by repeating the model-building procedure within each bootstrap sample, including univariable screening at P < 0.05 and backward stepwise elimination with P < 0.05 as the retention criterion, starting from all candidate predictors evaluated in the original primary analysis. The model selected in each bootstrap sample was evaluated in both that bootstrap sample and the original dataset. Optimism was calculated as the difference between these two AUCs. The mean optimism was then subtracted from the apparent AUC to obtain the optimism-corrected AUC. The 95% bootstrap interval was calculated using the 2.5th and 97.5th percentiles of the bootstrap distribution of the optimism-corrected AUC estimates.

A simple integer risk score was derived from the regression coefficients of the final model: snoring or OSA was assigned 2 points, reflecting its approximately twofold larger coefficient relative to the other predictors, while neck circumference ≥35 cm, Mallampati class III–IV, and absence of ≥2 upper incisors were each assigned 1 point, yielding a total score of 0–5. Model calibration was evaluated using the Hosmer–Lemeshow goodness-of-fit test. A two-sided P-value <0.05 was considered statistically significant.

## Results

### Study population

During the study period, 295 patients were screened for eligibility. Of these, 29 patients were excluded due to cancellation of surgery (n = 15), requirement for rapid sequence induction (n = 10), or conversion to regional anesthesia or monitored anesthesia care (n = 4). A total of 266 patients were included in the final analysis (Fig 1).

**Fig 1:**
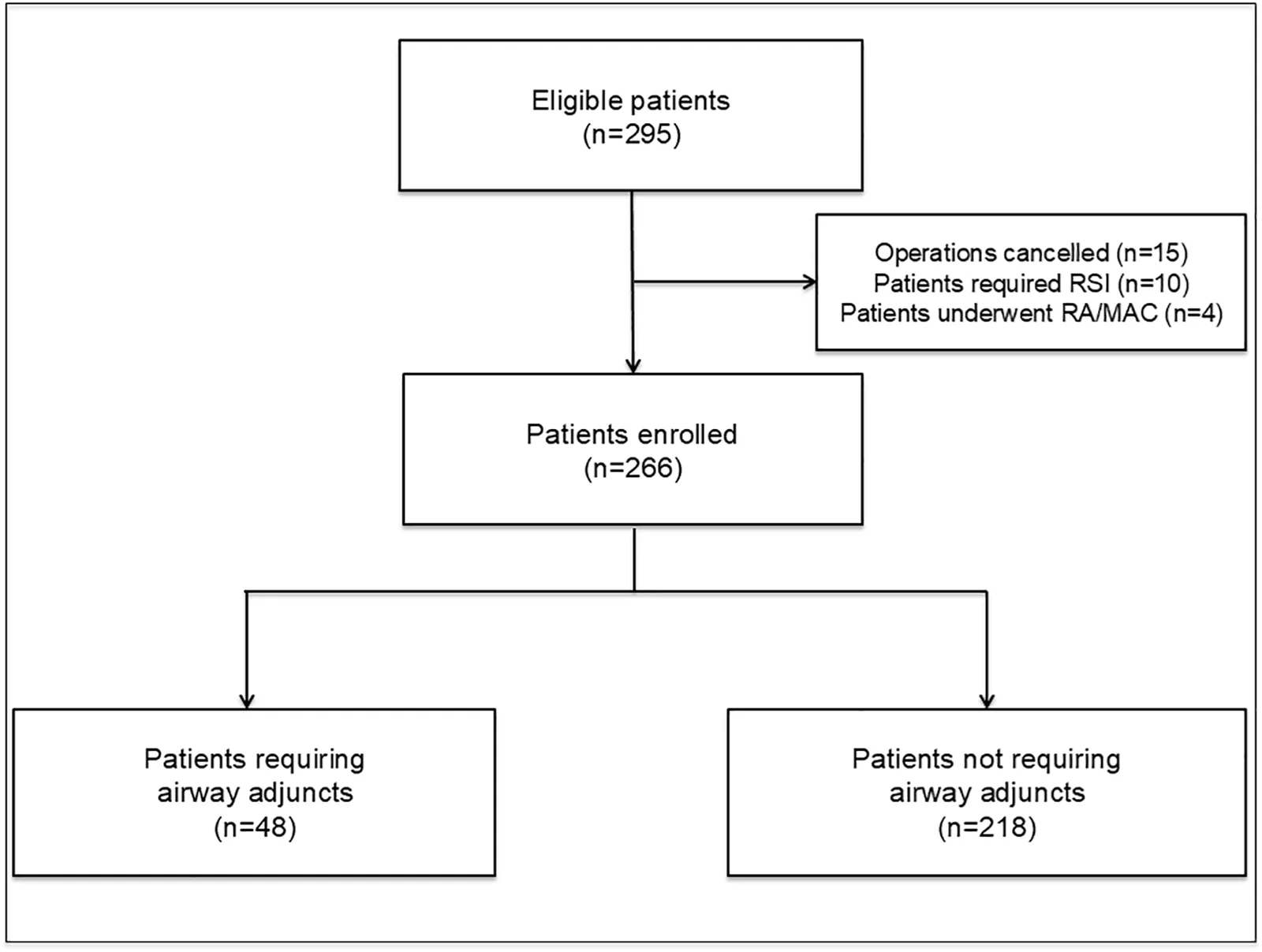
Study flow diagram of patient inclusion and analysis. RSI: Rapid sequence induction RA: Regional anesthesia MAC: Monitored Anesthesia Care.

### Baseline characteristics

The baseline characteristics of the study population are presented in Table 2. The mean age was 52.2 ± 14.5 years, 62.0% of participants were female, and the median body mass index (BMI) was 24.0 kg/m² (IQR, 21.3–27.0). Most participants were classified as American Society of Anesthesiologists (ASA) physical status II (69.9%).

**Table 2:** Baseline characteristics of the study population.

Variable	Total (n = 266)
Age (year), mean ± SD	52.2 ± 14.5
Female sex, n (%)	165 (62.0)
BMI (kg/m^2^), median (IQR)	24 (21.3–27.0)
ASA physical status, n (%)	
**-** Class I	16 (6.0)
**-** Class II	186 (69.9)
**-** Class III	64 (24.1)
Mask ventilation provider, n (%)	
- Anesthesiologist	28 (10.5)
− 2^nd^ year resident	70 (26.3)
− 3^rd^ year resident	27 (10.2)
- Nurse anesthetist	141 (53.0)
Airway assessment variables	
Snoring or OSA, n (%)	158 (59.4)
Absence of ≥2 upper incisors, n (%)	56 (21.1)
Neck circumference (cm), median (IQR)	34.0 (32.0–37.0)
Neck circumference ≥35 cm, n (%)	116 (43.6)
Mallampati class III–IV, n (%)	95 (35.7)

Data are presented as mean ± SD, median (IQR), or number (%)

BMI = body mass index; ASA = American Society of Anesthesiologists

### Incidence of the need for airway adjuncts during mask ventilation

The need for airway adjuncts during mask ventilation (Han grade ≥2) occurred in 48 patients (18.0%). Of these 48 patients, 38 (79.2%; 14.3% of the total cohort) had Han grade 2 and 10 (20.8%; 3.8% of the total cohort) had grade 3. No grade 4 events were observed. In a sensitivity analysis using the conventional Han grade ≥3 definition, only 10 patients (3.8%) met the outcome. The small number of events and limited data within some predictor categories precluded reliable multivariable logistic regression. Therefore, the grade ≥3 analysis was limited to descriptive reporting.

The rate of the need for airway adjuncts during mask ventilation did not differ significantly by provider type (attending anesthesiologist 21.4% [6/28], 2nd-year resident 21.4% [15/70], 3rd-year resident 25.9% [7/27], and nurse anesthetist 14.2% [20/141]; Fisher’s exact test, P = 0.067). To address the potential influence of provider experience on outcome ascertainment, a sensitivity analysis was performed including provider type as a covariate in the multivariable logistic regression model. All four anatomical predictors remained independently significant after adjustment: snoring or OSA (OR 5.75, 95% CI 2.09–15.85, P = 0.001), absence of ≥2 upper incisors (OR 2.28, 95% CI 1.06–4.90, P = 0.034), neck circumference ≥35 cm (OR 2.98, 95% CI 1.38–6.40, P = 0.005), and Mallampati class III–IV (OR 2.48, 95% CI 1.21–5.07, P = 0.013). Provider type was not independently associated with the need for airway adjuncts during mask ventilation after adjustment (all P > 0.46). These findings confirm that the identified anatomical predictors are independent of operator experience level.

### Univariable analysis

On univariable analysis, several factors were significantly associated with the need for airway adjuncts during mask ventilation, including male sex, BMI ≥ 30 kg/m², ASA physical status III, history of snoring or obstructive sleep apnea (OSA), absence of ≥2 upper incisors, neck circumference ≥35 cm, and higher Mallampati classification (all P < 0.05) (Table 3).

**Table 3:** Univariable analysis of factors associated with the need for airway adjuncts during mask ventilation.

Variable	No airway adjuncts (n = 218)	Airway adjuncts required (n = 48)	Crude OR (95% CI)	P-value
Sex (male), n (%)	76 (34.9)	25 (52.1)	2.03 (1.08–3.82)	0.026
BMI ≥ 30 kg/m², n (%)	14 (6.4)	10 (20.8)	3.83 (1.59–9.27)	0.002
ASA physical status III, n (%)	47 (21.6)	17 (35.4)	2.00 (1.02–3.91)	0.042
ULBT class ≥2, n (%)	36 (16.5)	7 (14.6)	0.86 (0.36–2.09)	0.750
Snoring or OSA, n (%)	115 (52.8)	43 (89.6)	7.70 (2.94–20.19)	<0.001
Absence of ≥2 upper incisors, n (%)	38 (17.4)	18 (37.5)	2.84 (1.44–5.62)	0.002
Neck circumference (cm), median (IQR)	34 (32–36)	37.2 (34.9–40.2)	—	<0.001
Mallampati class III–IV, n (%)	68 (31.2)	27 (56.2)	2.84 (1.50–5.37)	0.001

**Abbreviations:** BMI, body mass index; ASA, American Society of Anesthesiologists; ULBT, upper lip bite test; OSA, obstructive sleep apnea.

Other airway assessment variables, including interincisor distance, thyromental distance, sternomental distance, neck mobility, and presence of beard, were not significantly associated with the need for airway adjuncts during mask ventilation (S1 Table).

### Multivariable analysis

Variables with P < 0.05 in univariable analysis (male sex, BMI ≥ 30 kg/m², ASA status III, snoring or OSA, absence of ≥2 upper incisors, neck circumference ≥35 cm, and Mallampati class III–IV) were entered into the initial multivariable logistic regression model. After backward stepwise elimination, four variables remained associated with the need for airway adjuncts during mask ventilation (Han grade ≥2): snoring or OSA (aOR 5.27, 95% CI 1.94–14.35; P = 0.001), absence of ≥2 upper incisors (aOR 2.30, 95% CI 1.08–4.89; P = 0.030), neck circumference ≥35 cm (aOR 3.02, 95% CI 1.42–6.44; P = 0.004), and Mallampati class III–IV (aOR 2.47, 95% CI 1.22–4.97; P = 0.012) (Table 4).

**Table 4:** Multivariable logistic regression analysis of factors associated with the need for airway adjuncts during mask ventilation.

Variable	Adjusted OR	95% CI	P-value
Snoring or OSA	5.27	1.94–14.35	0.001
Absence of ≥2 upper incisors	2.30	1.08–4.89	0.030
Neck circumference ≥35 cm	3.02	1.42–6.44	0.004
Mallampati class III–IV	2.47	1.22–4.97	0.012

**Abbreviations:** OR, odds ratio; CI, confidence interval; OSA, obstructive sleep apnea.

The Hosmer–Lemeshow goodness-of-fit test showed no evidence of poor model fit (P = 0.077). Multicollinearity was not evident, with VIF values of 1.12 for snoring or OSA, 1.06 for absence of ≥2 upper incisors, 1.17 for neck circumference ≥35 cm, and 1.04 for Mallampati class III–IV. Male sex, BMI ≥ 30 kg/m², and ASA physical status III were not retained in the final model after adjustment. For male sex and BMI specifically, this likely reflects overlap of their effects with those of neck circumference and snoring or OSA rather than problematic multicollinearity, as suggested by the low VIF values. In a further sensitivity analysis addressing the TRIPOD recommendation against univariable pre-screening, all classic airway assessment variables (thyromental distance, sternomental distance, interincisor gap, neck flexion, neck extension, and beard presence) were entered simultaneously with the four primary predictors in a single multivariable model (10 variables; EPV 4.8, noting this is below the recommended minimum and results should be interpreted with caution). All four primary predictors remained independently significant: snoring or OSA (OR 5.83, 95% CI 2.09–16.28, P = 0.001), absence of ≥2 upper incisors (OR 2.34, 95% CI 1.05–5.20, P = 0.037), neck circumference ≥35 cm (OR 2.99, 95% CI 1.37–6.52, P = 0.006), and Mallampati class III–IV (OR 2.89, 95% CI 1.33–6.32, P = 0.008). Among the classic predictors, thyromental distance (P = 0.900), sternomental distance (P = 0.517), interincisor gap (P = 0.459), neck extension (P = 0.250), and beard (P = 0.845) were all non-significant. Neck flexion showed a borderline association (OR 0.89 per degree, 95% CI 0.81–0.98, P = 0.019), suggesting that reduced neck flexion may contribute to the need for airway adjuncts during mask ventilation; however, given the low EPV in this extended model, this finding should be considered exploratory and requires confirmation in a larger study.

### Predictive performance

Receiver operating characteristic (ROC) analysis showed that neck circumference alone had moderate discriminative ability (AUC 0.74, 95% CI 0.65–0.82). The cohort-derived cutoff of ≥35 cm yielded a sensitivity of 0.75, specificity of 0.63, positive predictive value (PPV) of 0.31, and negative predictive value (NPV) of 0.92. Because this cutoff was derived and evaluated using the same dataset, its performance may be optimistic and requires external validation.

The final four-variable logistic regression model, comprising snoring or OSA, absence of ≥2 upper incisors, neck circumference ≥35 cm, and Mallampati class III–IV, had an apparent AUC of 0.804 (95% CI 0.745–0.862) (Fig 2). Bootstrap internal validation with 1,000 resamples, repeating the model-building procedure within each resample, yielded a mean optimism of 0.040 and an optimism-corrected AUC of 0.764 (95% bootstrap interval 0.708–0.820). The combined four-variable model demonstrated higher discrimination than neck circumference alone.

**Fig 2:**
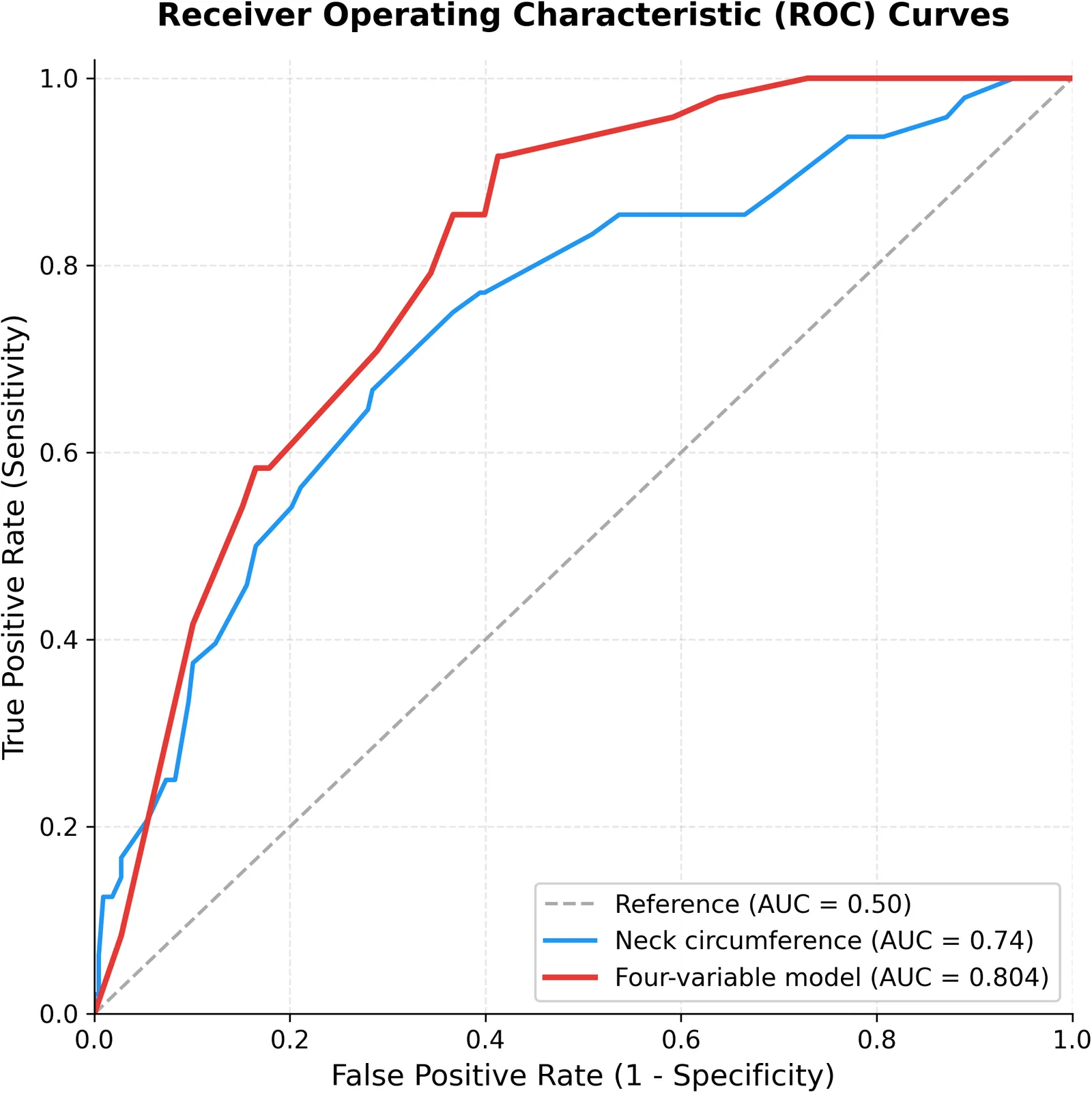
Receiver operating characteristic (ROC) curves for predicting the need for airway adjuncts during mask ventilation.

The blue curve shows neck circumference alone (AUC 0.74, 95% CI 0.65–0.82); the red curve shows the complete four-variable model combining snoring or OSA, neck circumference ≥35 cm, Mallampati class III–IV, and absence of ≥2 upper incisors (apparent AUC 0.804, 95% CI 0.745–0.862; optimism-corrected AUC 0.764, 95% bootstrap interval 0.708–0.820). The dashed diagonal line represents the reference line (AUC = 0.50).

To facilitate bedside application, a simple integer risk score was derived from the regression coefficients. Snoring or OSA was assigned a score of 2 (reflecting its approximately twofold larger coefficient relative to the other predictors), while neck circumference ≥35 cm, Mallampati class III–IV, and absence of ≥2 upper incisors were each assigned a score of 1. The total score ranges from 0 to 5.

The AUC of this integer score was 0.801, closely approximating the full logistic regression model (AUC 0.804). At a cutoff of ≥3, the score had a sensitivity of 0.85, specificity of 0.63, positive predictive value of 0.34, and negative predictive value of 0.95. Patients with a score of ≥3 (121/266, 45.5%) were classified as high risk for requiring airway adjuncts during mask ventilation. This score is presented as a hypothesis-generating tool requiring external validation before clinical adoption (Tables 5 and 6).

**Table 5:** Bedside risk score for predicting the need for airway adjuncts during mask ventilation.

Predictor	Score
Snoring or OSA	2
Neck circumference ≥35 cm	1
Mallampati class III–IV	1
Absence of ≥2 upper incisors	1
**Total possible score**	**0-5**

Score is derived from regression coefficients. OSA, obstructive sleep apnea.

High risk defined as score ≥3.

**Table 6:** Predictive performance of the bedside risk score at different cutoff values.

Score cutoff	Sensitivity	Specificity	PPV	NPV
≥1	1.00	0.24	0.23	1.00
≥2	0.96	0.41	0.26	0.98
≥3*	**0.85**	**0.63**	**0.34**	**0.95**
≥4	0.58	0.83	0.44	0.90
≥5	0.08	0.97	0.40	0.83

PPV, positive predictive value; NPV, negative predictive value.

* Exploratory cutoff (sensitivity 0.85, specificity 0.63).

## Discussion

In this prospective study, we identified four independent predictors of the need for airway adjuncts or optimization during mask ventilation among Thai adults undergoing elective surgery. The incidence was 18%, which is higher than figures reported in several earlier series. We attribute this primarily to our use of a broad definition (Han grade ≥2), which captures not only severe cases but also situations requiring airway adjuncts and thus may better reflect the spectrum of mask ventilation difficulty encountered in routine anesthetic practice.

Neck circumference stood out as a consistent and practically useful predictor. Its association with the need for airway adjuncts in the adjusted model was moderate, but what matters clinically is that it takes seconds to measure, requires no special equipment, and can be incorporated seamlessly into a routine preoperative visit. This is consistent with earlier reports and a recent meta-analysis by Hung et al. linking increased neck circumference to difficult airway management across diverse populations [10]. A plausible mechanism is that a larger neck circumference reflects excess peripharyngeal soft tissue, narrowing the upper airway and predisposing it to collapse once the patient is anesthetized [5].

Mallampati class III–IV was also an independent predictor. Although originally developed for laryngoscopy prediction, the link with mask ventilation is mechanistically straightforward: a crowded oropharynx can obstruct the upper airway once muscle tone is lost after induction of anesthesia. At the same time, it is important to recognize that Mallampati classification alone has limited predictive accuracy, as highlighted in a previous meta-analysis [11]. This reinforces the view that Mallampati class should be interpreted alongside other assessment variables, not in isolation.

Snoring or OSA was the strongest predictor in our model, with the highest adjusted OR (5.27). This is mechanistically plausible: patients with OSA have inherently collapsible upper airways and altered neuromuscular compensation, both of which are further compromised by anesthetic agents [5,15]. Earlier work has similarly linked sleep-disordered breathing to airway difficulty, especially when obesity or unfavorable anatomy is also present [15–17] However, several limitations of this exposure variable must be acknowledged. Snoring or OSA was ascertained by self-report using a single question, without a validated instrument or polysomnography confirmation. Self-reported snoring is non-specific and subject to recall bias; patients are frequently unaware of apneic episodes, and the 59.4% prevalence in this cohort may partly reflect this imprecision. Furthermore, snoring and diagnosed OSA were combined as a single dichotomous variable and cannot be separated retrospectively, which limits mechanistic interpretation. Structured screening tools such as the STOP-BANG questionnaire may provide more reliable and reproducible identification of OSA-related airway risk and should be considered in future studies.

The association between absence of ≥2 upper incisors and the need for airway adjuncts warrants clarification. It might be assumed that missing anterior teeth would facilitate mask ventilation by allowing the lips to collapse inward and improve mask contact; however, the mechanism may work in the opposite direction. Loss of upper incisor support causes the upper lip and perioral soft tissue to sink inward, creating an irregular facial contour that makes achieving an airtight mask seal difficult. Our findings are consistent with this explanation and with earlier reports, and serve as a reminder that dentition status should be explicitly assessed during preoperative airway evaluation [8].

Our results broadly agree with the existing literature while also adding a Southeast Asian perspective. Older studies tended to focus on anatomical predictors of difficult intubation [11,18], while more recent work has shifted attention toward anthropometric variables such as neck circumference for predicting difficult mask ventilation [9,17]. Notably, the optimal cutoff in our cohort (≥35 cm) was lower than thresholds commonly reported in Western studies: Kheterpal et al. reported a cutoff of ≥40 cm [17], and De Jong et al. reported >43 cm [19] as predictors of difficult airway. This difference may reflect the generally smaller body habitus in Thai patients — lower average BMI at equivalent levels of upper airway adiposity [13] and distinct fat deposition patterns around the neck and pharynx [14] — which may produce clinically meaningful airway compromise at lower absolute neck circumference values. This highlights an important consideration when applying published cutoffs: thresholds derived from predominantly Western cohorts should not be assumed to transfer directly to Southeast Asian populations, and population-specific calibration is warranted.

Taken together, these findings argue for a multi-variable approach to preoperative airway assessment. None of the four predictors performs well enough in isolation to be used alone, but combining all four yielded substantially better discrimination than any single predictor alone. To facilitate bedside use, we derived a simple integer risk score; at the exploratory cutoff of ≥3, the score achieved a high NPV of 0.95, suggesting that patients with a score below this threshold are unlikely to require airway adjuncts during mask ventilation. Importantly, all four predictors require nothing more than a focused history and a tape measure — resources available in virtually every anesthetic setting. This aligns with current guidelines, which call for anticipating difficulty in both ventilation and intubation as part of comprehensive airway planning [4].

It should be noted that this risk score predicts Han grade ≥2, representing the need for airway adjuncts or optimization during mask ventilation, and cannot be applied to predict grade ≥3 or grade 4 events, which represent more severe mask ventilation difficulty requiring two providers or impossible ventilation. The small number of grade ≥3 events (n = 10) in this cohort precluded separate modelling of this clinically more critical outcome.

Bootstrap internal validation yielded an optimism-corrected AUC of 0.764. Nevertheless, external validation across different clinical settings and patient populations is needed before either the model or the risk score can be recommended for broader clinical use [20,21].

### Strengths and limitations

This study has several strengths. First, the prospective design with airway assessments carried out in a standardized manner by a single evaluator helped minimize interobserver variability. Second, consecutive enrollment of eligible patients reduced the risk of sampling bias. Third, collecting a wide panel of airway variables allowed comprehensive evaluation of multiple candidate predictors rather than testing only pre-specified factors. Fourth, two post-hoc sensitivity analyses (one adjusting for provider experience and the other simultaneously including the classic airway assessment variables) both supported the robustness of the primary findings. Fifth, bootstrap internal validation with 1,000 resamples, repeating the model-building procedure within each resample, provided an optimism-corrected AUC of 0.764 (95% bootstrap interval 0.708–0.820). Finally, this study addresses an important evidence gap by providing prediction model data from a Southeast Asian cohort, where anthropometric characteristics differ meaningfully from predominantly Western reference populations.

Several limitations deserve acknowledgement. First, this was a single-center study at a university teaching hospital in Southern Thailand, and the findings may not generalize to hospitals with different patient demographics or anesthetic practices. Second, Han grade was assigned by the primary ventilating provider (self-grading) without an independent observer, which may have introduced subjective bias, particularly for junior providers who may apply different thresholds when escalating to airway adjuncts. Third, our use of Han grade ≥2 is broader than the grade ≥3 threshold used in most reference studies (incidence 1.4–7.8%); only 10 grade-3 events occurred, precluding multivariable analysis under the stricter definition. The model therefore predicts the need for airway adjuncts or additional optimization rather than more severe mask ventilation difficulty, and cannot be applied to grade ≥3 or grade 4 events; the clinical significance of a positive score should be interpreted accordingly. Fourth, the variable selection strategy (univariable screening at P < 0.05 followed by backward elimination) introduces selection bias and may have excluded predictors whose effects were masked in univariable analysis; a sensitivity analysis including all classic predictors a priori confirmed the four primary predictors remained significant, and neck flexion showed a borderline association (OR 0.89, P = 0.019) that warrants confirmation in larger studies. Fifth, although the EPV of 12:1 meets the recommended minimum, the relatively modest number of outcome events (n = 48) means that odds ratio estimates, particularly for snoring or OSA with its wide confidence interval, should be interpreted with appropriate caution. Sixth, excluding patients with known difficult airways or requiring rapid sequence induction introduces selection bias; the true incidence and predictor weights in an unselected clinical population may be higher than reported here. Finally, we did not assess difficult laryngoscopy or intubation separately, so we cannot comment on combined difficult airway scenarios.

## Conclusion

In this cohort of Thai adults undergoing elective general anesthesia, four bedside variables—neck circumference ≥35 cm, Mallampati class III–IV, absence of ≥2 upper incisors, and a history of snoring or OSA—were independently associated with the need for airway adjuncts during mask ventilation. These predictors are not novel; they correspond closely to the structural components of established Western frameworks including the Langeron criteria [8] and the MOANS mnemonic (Mask seal, Obesity/Obstruction, Age > 55, No teeth, Stiff/Snoring) [22]. The principal contribution of this study is to validate these risk factors in a Southeast Asian cohort and to demonstrate that a population-specific neck circumference threshold (≥35 cm, lower than the ≥ 40 cm typically used in Western populations) may be more appropriate for Thai patients. A simple bedside integer risk score combining these predictors demonstrated good discrimination (AUC 0.801), closely approximating the full logistic regression model (AUC 0.804). As a single-center derivation study with a modest number of events, these findings are hypothesis-generating and require external validation in independent Southeast Asian cohorts before clinical adoption.

## Supporting information

S1 Table Full airway assessment variables and their association with the need for airway adjuncts. Variables not significantly associated with the need for airway adjuncts in univariable analysis are listed. Data are presented as median (IQR) or number (%).
(DOCX)

S1 Checklist STROBE Statement—checklist of items that should be included in reports of observational studies. (DOCX)
