# Supplementary material for: Predictors of the need for airway adjuncts during mask ventilation in Thai adult patients undergoing general anesthesia: A prospective observational study
Source: PLoS One. 2026 Oct 6;21(10):e0359773. doi: 10.1371/journal.pone.0359773 (PMC13641351; doi:10.1371/journal.pone.0359773)
Supplement: Supplementary file 1 [file pone.0359773.s001.docx]

Predictors of difficult mask ventilation in Thai adult patients undergoing general anesthesia: A prospective observational study

**S1 Table.** Full airway assessment variables and their association with the need for airway adjuncts during mask ventilation.

| **Variable** | **No airway adjuncts (n=218)** | **Airway adjuncts required (n=48)** | **P-value** |
| --- | --- | --- | --- |
| Interincisor distance (cm) | 6 (5.5–6.5) | 6 (5.5–6.5) | 0.584 |
| Thyromental distance (cm) | 9 (8.5–10) | 9 (9–10) | 0.618 |
| Sternomental distance (cm) | 18 (17–19) | 18 (17–19) | 0.905 |
| Neck flexion (degrees) | 45 (45–48) | 45 (40–45) | 0.057 |
| Neck extension (degrees) | 50 (45–50) | 50 (45–55) | 0.720 |
| Presence of beard, n (%) | 10 (4.6) | 5 (5.6) | 0.158 |

*Data are presented as median (IQR) or number (%).*
