# Supplementary material for: Predictors of the need for airway adjuncts during mask ventilation in Thai adult patients undergoing general anesthesia: A prospective observational study
Source: PLoS One. 2026 Oct 6;21(10):e0359773. doi: 10.1371/journal.pone.0359773 (PMC13641351; doi:10.1371/journal.pone.0359773)
Supplement: Supplementary file 2 [file pone.0359773.s002.docx]

**STROBE Statement — Checklist of items that should be included in reports of observational studies**

*Study type: Prospective observational (cohort) study*

*Manuscript: Predictors of Difficult Mask Ventilation in Adult Patients Undergoing General Anesthesia: A Prospective Observational Study*

| **Item** | **No** | **Recommendation** | **Reported on page/section** |
| --- | --- | --- | --- |
| **TITLE AND ABSTRACT** | | | |
| Title and abstract | 1 | (a) Indicate the study's design with a commonly used term in the title or the abstract (b) Provide in the abstract an informative and balanced summary of what was done and what was found | Title; Abstract |
| **INTRODUCTION** | | | |
| Background/rationale | 2 | Explain the scientific background and rationale for the investigation being reported | Introduction, para 1–3 |
| Objectives | 3 | State specific objectives, including any pre-specified hypotheses | Introduction, last para |
| **METHODS** | | | |
| Study design | 4 | Present key elements of study design early in the paper | Methods: Study design |
| Setting | 5 | Describe the setting, locations, and relevant dates, including periods of recruitment, exposure, follow-up, and data collection | Methods: Study design (Songklanagarind Hospital; Sep 2018 – Oct 2019) |
| Participants | 6 | (a) Give the eligibility criteria, and the sources and methods of selection of participants (b) Describe methods of follow-up (cohort) / matching criteria (case-control) | Methods: Participants |
| Variables | 7 | Clearly define all outcomes, exposures, predictors, potential confounders, and effect modifiers. Give diagnostic criteria, if applicable | Methods: Airway assessment; Outcome |
| Data sources/ measurement | 8 | For each variable of interest, give sources of data and details of methods of assessment (measurement). Describe comparability of assessment methods if there is more than one group | Methods: Airway assessment |
| Bias | 9 | Describe any efforts to address potential sources of bias | Methods: Study design; Discussion: Limitations |
| Study size | 10 | Explain how the study size was arrived at | Methods: Sample size |
| Quantitative variables | 11 | Explain how quantitative variables were handled in the analyses. If applicable, describe which groupings were chosen and why | Methods: Statistical analysis |
| Statistical methods | 12 | (a) Describe all statistical methods, including those used to control for confounding (b) Describe any methods used to examine subgroups and interactions (c) Explain how missing data were addressed (d) If applicable, describe analytical methods taking account of sampling strategy (e) Describe any sensitivity analyses | Methods: Statistical analysis |
| **RESULTS** | | | |
| Participants | 13 | (a) Report numbers of individuals at each stage of study (b) Give reasons for non-participation at each stage (c) Consider use of a flow diagram | Results, para 1; Fig. 1 |
| Descriptive data | 14 | (a) Give characteristics of study participants (b) Indicate number of participants with missing data for each variable of interest | Results; Table 1 |
| Outcome data | 15 | Report numbers of outcome events or summary measures | Results; Tables 2–3 |
| Main results | 16 | (a) Give unadjusted estimates and, if applicable, confounder-adjusted estimates (b) Report category boundaries when continuous variables were categorized (c) If relevant, consider translating estimates of relative risk into absolute risk for a meaningful time period | Results; Tables 2–3 |
| Other analyses | 17 | Report other analyses done—e.g. analyses of subgroups and interactions, and sensitivity analyses | Results; Fig. 2 (ROC analysis) |
| **DISCUSSION** | | | |
| Key results | 18 | Summarize key results with reference to study objectives | Discussion, para 1 |
| Limitations | 19 | Discuss limitations of the study, taking into account sources of potential bias or imprecision. Discuss both direction and magnitude of any potential bias | Discussion: Strengths and Limitations |
| Interpretation | 20 | Give a cautious overall interpretation of results considering objectives, limitations, multiplicity of analyses, results from similar studies, and other relevant evidence | Discussion |
| Generalisability | 21 | Discuss the generalisability (external validity) of the study results | Discussion: Strengths and Limitations |
| **OTHER INFORMATION** | | | |
| Funding | 22 | Give the source of funding and the role of the funders for the present study and, if applicable, for the original study on which the present article is based | Declarations: Funding (No specific funding) |

** An Explanation and Elaboration article discusses each checklist item and gives methodological background and published examples of transparent reporting. The STROBE checklist is best used in conjunction with this article (freely available on the Web sites of PLoS Medicine at http://www.plosmedicine.org/, Annals of Internal Medicine at http://www.annals.org/, and Epidemiology at http://www.epidem.com/). Information on the STROBE Initiative is available at www.strobe-statement.org.*
